# Xanthan Gum-Driven Innovations for Reinventing Food Preservation

**DOI:** 10.3390/polym17233160

**Published:** 2025-11-27

**Authors:** Zeba Tabassum, Abhinav Anand, Rishab Bhanot, Madhuri Girdhar, Anil Kumar, Narsimha Mamidi, Anand Mohan

**Affiliations:** 1 School of Bioengineering and Biosciences, Lovely Professional University, Phagwara 144401, Punjab, India; zebatabassum326@gmail.com; 2 University School of Pharmaceutical Sciences, Rayat Bahra Professional University, Hoshiarpur 146101, Punjab, India; dr.abhinav.anand@rayatbahra.com (A.A.); rishab.bhanot@rayatbahra.com (R.B.); 3 Division of Research and Development, Lovely Professional University, Phagwara 144401, Punjab, India; madhuri.23745@lpu.co.in; 4 Gene Regulation Laboratory, National Institute of Immunology, New Delhi 110067, Delhi, India; anilk@nii.ac.in; 5 Wisconsin Centre for Nano Biosystems, School of Pharmacy, University of Wisconsin-Madison, Madison, WI 53705, USA

**Keywords:** biopolymers, xanthan gum, blend, edible coating, sustainability

## Abstract

Continuous anthropogenic inputs have raised environmental concerns regarding non-degradable plastics derived from non-renewable petrochemicals, creating an urgent need for sustainable alternatives and driving a paradigm shift toward bioplastics. This review investigates the transformative role of the natural biopolymer xanthan gum as an eco-friendly additive in advancing biodegradable materials. Derived from *Xanthomonas campestris*, xanthan gum offers non-toxicity, biodegradability, and strong compatibility. The literature indicates that its negative charge enables interactions with positively charged molecules, enhancing composite properties such as mechanical strength. Although xanthan gum has limitations when used alone, it functions as an effective additive in packaging applications. The novelty of this work lies in exploring diverse techniques and formulations for integrating xanthan gum into bioplastic films and coatings, emphasizing its role in reinforcing biopolymer structures. As a sustainable alternative, xanthan gum-based composites preserve food quality and extend shelf life by providing protection against moisture, oxygen, UV radiation, and microbial contamination. Realizing its full potential requires optimized formulations to prevent structural disruptions and reduced stretchability at higher xanthan gum concentrations. Continued research, especially leveraging nanotechnology, is essential to amplify its advantages and address related challenges. This review highlights xanthan gum’s pivotal contribution to bioplastic innovation, presenting a strong case for its broader adoption in the food packaging industry.

## 1. Introduction

Packaging materials play a crucial role in ensuring the protection of the products throughout their journey from production to distribution. The extensive use of traditional packaging has contributed to the global economy’s growth, facilitating secure and efficient transportation. Plastic, with its unparalleled attributes, has emerged as the preferred choice among conventional packaging materials. Its versatility, durability, light weight, and cost-effectiveness make it indispensable in various industries, dominating our daily lives for over six decades. However, the widespread disposal of non-biodegradable plastic waste over the years has led to adverse environmental consequences. This calls for a re-evaluation of our reliance on plastics and an exploration of sustainable alternatives to mitigate the environmental impact of packaging materials. Adopting sustainable alternatives can cut carbon emissions, lessen pollution, and protect ecosystems, encouraging a more resilient and environmentally friendly approach to packaging solutions [1,2,3,4,5,6,7,8,9].

One type of green asset that has the capability to substantially replace synthetic polymers and their negative attributes is biopolymers and their composites. Biopolymers and biopolymer-based composites, which offer biodegradability, renewability, and compatibility with various industrial and biomedical applications, have demonstrated their effectiveness across various applications, including food packaging [10], tissue engineering [11,12,13], carcinogenesis treatment [14], effluent remediation [15], etc., demonstrating their multifunctionality and ecological benefits. As biotechnology and material science research develops, biopolymers have the potential to replace synthetic polymers and contribute to a future that is more ecologically friendly fostering circular. The continuous study in this area has enormous promise for promoting sustainable growth and reducing the environmental impact of contemporary businesses and technologies [4,5,16,17].

**Figure 1:**
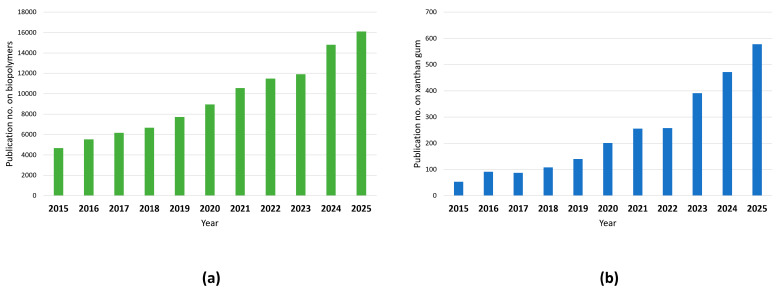
Increasing trend in the number of research publications on (**a**) biopolymers and (**b**) xanthan gum in recent years (https://www.sciencedirect.com/search, accessed on 18 October 2025).

**Figure 2:**
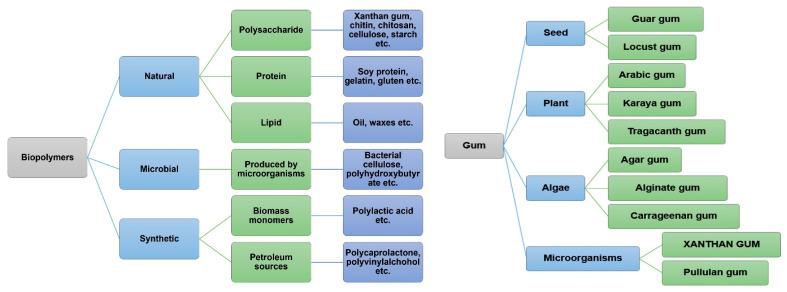
Sources of biopolymers and natural gums [24,25].

Xanthan gum is a biopolymer that has garnered significant interest recently from scientists all over the world due to its versatile characters, including high viscosity, stability across a range of conditions, and excellent gelling properties. An upward trend in research publications on biopolymers and xanthan gum has been observed in recent years, with Figure 1 and Figure 2, respectively, illustrating this growth and the wide range of biopolymers and bio-based gums currently under investigation. Various research efforts are focused on enhancing its efficiency in applications [18], reducing production costs [5], and expanding its applicability across numerous industries that include food [19], pharmaceuticals [20], cosmetics [21], packaging [3], waste water treatment [22], etc. Additional applications can be found in construction engineering and energy industry, particularly for oil extraction and lubrication during mud drilling [23]. The development of such materials aligns closely with several United Nations Sustainable Development Goals (SDGs), notably SDG 9 (Industry, Innovation, and Infrastructure), SDG 12 (Responsible Consumption and Production), and SDG 13 (Climate Action). SDG 9 emphasizes sustainable industrialization and innovation, encouraging the transition toward circular and resource-efficient manufacturing systems through the adoption of green materials. SDG 12 promotes waste minimization and resource optimization by transforming agro-industrial residues into value-added biopolymers, thereby supporting closed-loop production systems. SDG 13 underscores the necessity of reducing greenhouse gas emissions and mitigating climate change impacts, which can be achieved through the substitution of conventional plastics with biodegradable and renewable materials.

The paper provides a comprehensive analysis of recent advancements and applications of xanthan gum in the development of sustainable food packaging materials. It particularly emphasizes xanthan gum’s role in enhancing the structural, mechanical, and barrier properties of bioplastic and edible coating formulations. Current advancements in the formulation, modification, and performance enhancement of xanthan gum-based materials are also discussed. The study emphasizes xanthan gum’s role in improving the structural, mechanical, and barrier properties of bioplastics and coatings, underscoring its transformative potential in developing next-generation, eco-friendly packaging systems for a sustainable future.

## 2. Xanthan Gum: A Green Polymer

**Figure 3:**
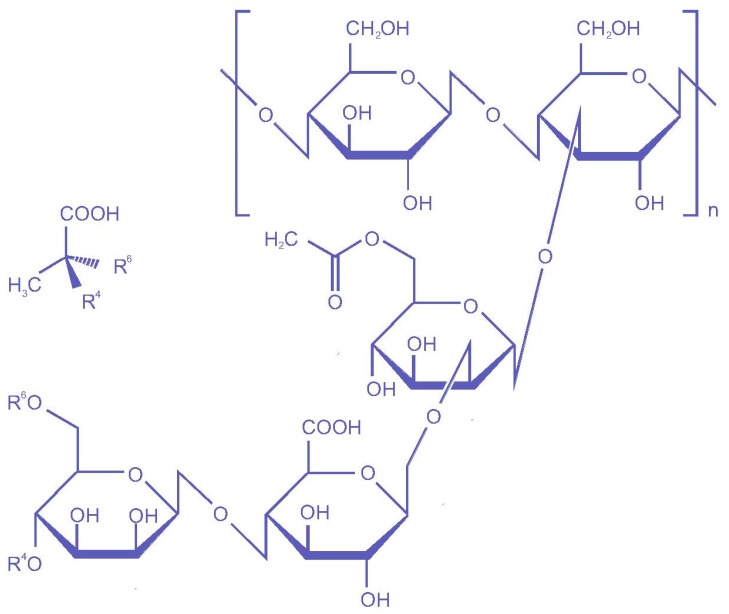
Chemical structure of xanthan gum.

**Figure 4:**
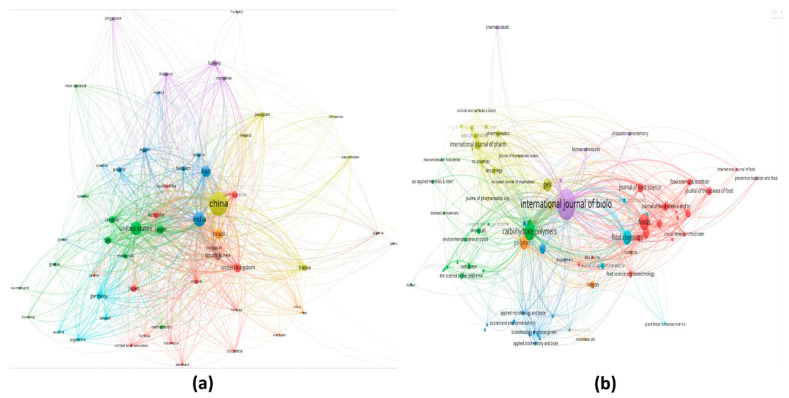
Emerging research trends on xanthan gum-based packaging and coating: (**a**) country-wise distribution and (**b**) top journals publishing related studies [Bibliometric analysis was performed by using Dimensions AI|The most advanced scientific research database and VOSviewer—Visualizing scientific landscapes].

Xanthan gum (C_35_H_49_O_29_)_n_ is one of these gums, a microbial polysaccharide (branched heteropolysaccharide), typically produced by *X. campestris* (Gram-negative bacterium) during the fermentation process. It is a high molecular weight (2 × 10^6^ to 10^7^) biopolymer, composed of pentasaccharide units (2:2:1 molar ratio of D-glucose, D-mannose, and D-glucuronic acid residues), which was discovered by Allene Jeanes in 1950. Its chemical composition closely resembles cellulose, consisting of β-D-glucose residues connected by 1,4 bonds. The primary chains contain a trisaccharide side chain, comprising β-D-mannose-β-D-glucuronic acid-α-D-mannose, linked to alternating D-glucose units in the main chain through α-1,3 linkages [26]. The chemical structure of the xanthan gum is shown in Figure 3. Due to the presence of glucuronic acid, xanthan gum is anionic, which contributes to its ability to interact with other cations and positively charged molecules. Since 1969, xanthan gum has been employed as a food additive, and it is regarded as an important natural and industrial biopolymer, due to its remarkable qualities, including non-toxicity, the capacity for cell proliferation, and its non-sensitizing effect [25]. It is a safe food additive, approved by the US FDA (United States Food and Drug Administration) and registered as a stabilizer and emulsifier in the CFR (Code of Federal Regulations). The xanthan gum market is expected to grow at the rate of 4.5% between 2019 and 2024 and will reach a valuation of USD 1.2 billion [27]. Its biodegradability and compatibility make it suitable for its application in food packaging in the form of films or coatings [3,26,28]. Figure 4 depicts emerging research trends on xanthan gum-based packaging and coating, highlighting country-wise distribution and leading journals. The bibliometric analysis was performed using Dimensions and VOSviewer software to plot the graphs.

**Figure 5:**
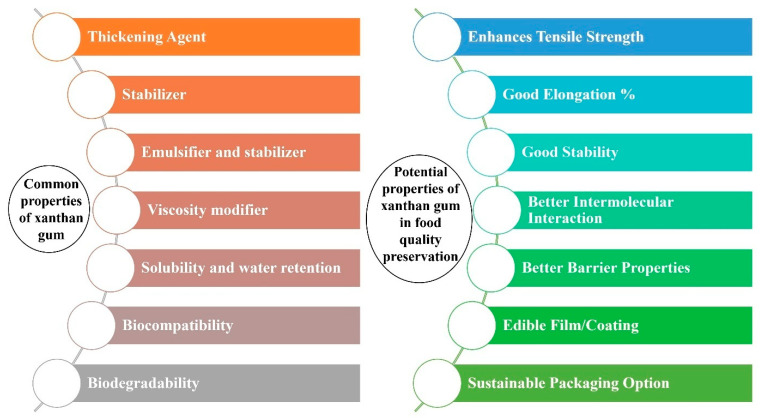
Properties of xanthan gum and xanthan gum-based composites.

Current research on xanthan gum for packaging and coating applications focuses on its role as a functional additive to overcome limitations in existing bioplastic-based food packaging and improve their commercial viability. Additionally, producing xanthan gum from waste-derived substrates presents new opportunities for sustainable, low-cost, and circular bio-based packaging solutions. Xanthan gum offers several benefits compared to other natural gums in packaging applications. It has exceptional rheological properties, providing stable viscosity across a wide range of temperature and pH level, which ensures consistent performance in various environmental conditions. Its strong film-forming ability and compatibility with other biopolymers enable the creation of robust, flexible, and transparent films ideal for food packaging. Xanthan gum has the ability to enhance stability when added with other polymers [29]. Additionally, xanthan gum enhances the mechanical strength and barrier properties of composite films, reducing permeability to gases and moisture, which is crucial for extending the shelf life of perishable goods. Its biodegradability and non-toxicity further align with the growing demand for sustainable and environmentally friendly packaging solutions. Properties of xanthan gum and xanthan gum-based composites are shown in Figure 5.

## 3. Sustainable Origins for Xanthan Gum: Exploring Environment Friendly Sources

To overcome the high cost associated with synthesis of xanthan gum from synthetic media, various efforts have been directed towards utilizing various waste sources [5,30,31]. The choice of cultivation medium is crucial in the production of this biopolymer. Numerous efforts have been made to produce xanthan gum using agriculture-based and food industry wastes as alternatives to synthetic media, which can be costly. Achieving an economically viable manufacturing process also involves optimizing the composition and processing parameters. Several strategies have been explored, including adjusting nutrient content and feeding methods, controlling temperature and pH, optimizing agitation, and incorporating antifoam agents in xanthan gum fermentations. A recent publication describes different waste materials that are a potential source of xanthan gum production with a sustainable approach [5].

A team of researchers has recommended further investigation into the potential of novel microbial strains to enhance xanthan gum production. The isolation and characterization of these new strains hold the promise of developing more efficient and cost-effective processes for xanthan production. Additionally, exploring genetic engineering techniques is an avenue worth pursuing to modify its properties. The advancement of fermentation techniques and bioreactors can play a crucial role in improving production. Optimization of fermentation parameters, such as temperature, pH, and nutrient supplementation, can contribute to enhancing the overall efficiency. Furthermore, the implementation of advanced monitoring and control systems has the potential to improve the consistency and efficiency of the fermentation process [23].

Xanthan gum was synthesized by *X. campestris* pv. *campestris* strains 1866 and 1867 using lignocellulosic agro-industrial residues. Xanthan gum was produced through agitation on an orbital shaker, utilizing a culture medium containing coconut shell, cocoa husks, or sucrose, with minimal supplementation of urea and potassium. The production varied among the substrates, with higher yields observed with cocoa husks for both strains (4.48 g L^−1^ for 1866 and 3.89 g L^−1^ for 1867). It also exhibited favorable thermal stability [32].

Some researchers developed a modified bacterium called *Sphingomonas sanxanigenens* NXG-P916 to efficiently produce xanthan gum using corn straw. They engineered this strain by introducing a xanthan gum production module into the genome of a base strain (NXdPE). This base strain was capable of generating activated precursors of polysaccharides. They optimized the expression of xanthan gum genes by using a suitable promoter, P916. The resulting strain, NXG-P916, produced 9.48 g/kg of xanthan gum when using glucose as a carbon source, a 2.1-fold improvement over the original engineered strain. In batch fermentation using corn straw hydrolysate containing both glucose and xylose, NXG-P916 produced 12.72 g/kg of xanthan gum with an extremely high molecular weight, showing promising potential for producing high-quality xanthan gum from renewable resources. The base strain, NXdPE, holds promise as a platform for producing polysaccharides from biomass hydrolysates [33]. In the fermentative process, the carbon source accounts for a significant portion of production costs, prompting the exploration of cost-effective and sustainable alternatives. Utilizing agricultural residues like corncobs, which are abundant, is particularly appealing. A study by Meirielly and colleagues aimed to assess the potential of hemicellulose fractions derived from alkaline-extracted corncobs as a carbon source for *Xanthomonas* sp. strains (629, 1078, 254, and S6) in xanthan gum production. Their findings revealed that strain 629 yielded the highest production (8.37 ± 5.75 g L^−1^) when utilizing a fermentation medium comprising saccharose (1.25%), hemicellulose fractions (3.75%), and salts. In the same medium, strain 629 produced gum with superior properties, including higher apparent viscosity (9298 ± 31 mPa s^−1^) at a shear rate of 10 s^−1^ and 25 °C in a 3% aqueous solution [34]. It is important to note that while these sources have potential, research is ongoing to optimize production processes, improve yields, and ensure economic viability. The choice of a green source depends on factors such as availability, cost-effectiveness, and environmental impact. Additionally, regulatory considerations and consumer preferences for sustainably sourced ingredients play a role in the adoption of alternative sources for xanthan gum production.

## 4. Advancement in Enhancing Overall Characteristics of Xanthan Gum-Based Packaging and Coating

**Figure 6:**
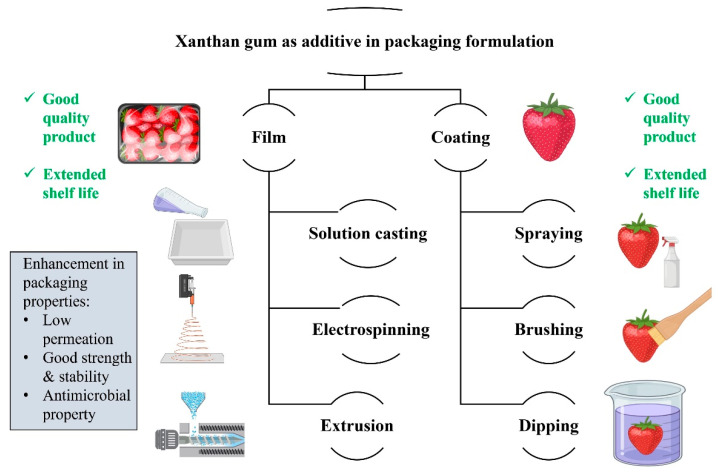
Xanthan gum in packaging formulation to achieve enhanced properties through different techniques.

A wide range of advanced techniques including solution casting, electrospinning, emulsion-based formulation, crosslinking, and nanocomposite integration are being employed to harness xanthan gum’s functional potential in packaging materials. These approaches enable tailored improvements in mechanical strength, barrier properties, and active functionality, thereby enhancing the overall performance of biopolymer-based packaging systems. Figure 6 illustrates the diverse techniques employed to leverage xanthan gum in packaging formulations, enhancing their properties. Using the casting method, researchers developed chitosan–xanthan gum composite films that demonstrated enhanced thermal stability. In thermogravimetric analysis, pure chitosan retained 30.406 wt% at 498.99 °C, whereas the chitosan–xanthan gum composite retained 45.74 wt%, indicating a significant widening of the thermal stability window. Films with higher xanthan gum content also exhibited superior tensile strength, confirming the reinforcing effect of xanthan gum in the composite structure [3]. However, incorporating xanthan gum did not alter the films’ solubility, moisture content, or water vapor permeability [29]. Blending chitosan with xanthan gum produced a slight improvement in tensile strength, accompanied by reduced flexibility, likely due to polyelectrolyte complex formation. Despite this loss in flexibility, the interaction enhanced overall durability, hydrophobicity, and barrier performance, including resistance to gases, water vapor, and UV radiation. However, the combination did not yield any notable gains in thermal stability [3].

Foamlike aerogel composites were made by Wang et al. using xanthan gum, sodium montmorillonite clay, and agar through an eco-friendly freeze-drying method. Fourier transform infrared spectroscopy (FTIR) confirmed molecular interactions among the materials that further improved mechanical properties and thermal stability significantly. Xanthan gum-clay aerogels had lower flammability due to clay’s flame retardancy effect, improving heat and mass transport [35].

Xanthan gum and TiO_2_-Ag nanoparticles were used by a group of scientists to modify the lemon peel powder film. Findings suggested that the film’s solubility and water vapor permeability dropped, whereas its tensile strength and Young’s modulus both remarkably rose (*p* ≤ 0.05). The film appeared as homogenous, granular film in the scanning electron micrographs. It also demonstrated greater thermal stability than the control sample, as per the thermal analysis data. The X-ray diffraction analysis (XRD) analysis results demonstrated that the crystalline characteristics of the films were decreased by the addition of TiO_2_&Ag nanofillers and xanthan gum. Additionally, it proved to have significant antibacterial action [36].

In another study, xanthan gum and hydroxypropyl methylcellulose were blended to synthesize a new packaging film. Good compatibility was achieved between the two materials with hydrogen bond interaction. Xanthan gum also possessed considerable impact on its chemical makeup, crystalline texture, and microstructure of the composite film. By coating bananas with the best sample, which had an ideal xanthan gum concentration of 2 g/L, the weight loss rate on bananas was reduced from 25 ± 3% (without coating) to 16 ± 4% (with coating). As a result, there was a reduction in the release of flavoring agents. Utilizing this composite film for food preservation has increased the quality of banana shelf life [37].

Chen and team fabricated composite films comprising polyvinyl alcohol and xanthan gum with good degrading qualities, using a casting technique. Compared to the pure polyvinyl alcohol films, the addition of xanthan gum was able to reduce moisture content, water solubility, and water vapor permeability. This material has the finest mechanical qualities as well. Furthermore, it showed greater food packaging capabilities than commercial plastic bags. Moreover, the packaging completely dissolves and degrades in soil and water in only about 12 h, which is much quicker than commercial plastic bags and even other biodegradable materials [38].

Combining polysaccharides like pullulan and xanthan gum with grape seed extract proved to improved mechanical characteristics and demonstrated inhibitory effects against various microbial strains. Because the composite film was able to slow down weight loss and maintain more vitamin C and total polyphenol even on the fifth day, it was successful in retaining 91% of the vitamin C and 72% of the total polyphenols, respectively. On the basis of the findings, investigators declared that this is a potential material to prolong shelf life of fresh-cut apples [39].

A new film was created by researchers using extracts from *Polygonatum cyrtonema*, xanthan gum, flaxseed gum, and carboxymethyl cellulose. Structural investigations through FTIR and XRD revealed that all the components interacted via hydrogen bonds. It was discovered that the best ratio film with the lowest water vapor transmission rate was able to extend the shelf life of mango when compared to the control sample. After 8 days of storage, the mango’s overall quality was enhanced by having a lower decay rate, weight loss rate, total soluble solid, and polyphenol oxidase, as well as a higher titratable acidity, and superoxide dismutase level than control mango [40].

Electrospun fibers made of gelatin are a potential material for food packaging, but they have poor mechanical qualities and high hydrophilicity. So, Yavari and colleagues used gelatin-based nanofibers and strengthened them utilizing oxidized xanthan gum as a crosslinking agent to get over these drawbacks. Through SEM analysis of the nanofibers’ morphology, it was discovered that by increasing xanthan gum concentration, the diameter of the fibers was reduced. The ideal sample obtained demonstrated a tensile stress of 13.24 ± 0.76 MPa, which is up to 10 times more than pure gelatin fiber. Furthermore, the resultant fibers with more oxidized xanthan gum content displayed significant tensile stress. Xanthan gum addition to the gelatin fibers increased heat stability and porosity while decreasing water vapor permeability, water solubility, and moisture content. Additionally, the propolis containing nanofibers had a uniform shape, posing high antioxidant and antibacterial activities [41].

With varying weight percentages of agar and xanthan gum, binary composite films were created by Rumanikrishnan and coworkers. The composite film was transparent, light, and biodegradable. By using the Fourier transform infrared spectroscopy, X-ray powder diffraction, and scanning electron microscopy techniques, the structure and shape of the produced composite film were verified. When compared to neat agar, composite films’ glass transition temperature (T_g_) and melting temperature (T_m_) were found to be slightly higher. The char yield was significantly increased, and the thermal stability had enhanced, according to thermogravimetric analysis (TGA) results. However, the water vapor permeability value reduced in the composite films and the difference was not significant [42].

Hydrocolloid-based biodegradable film with incorporation of oil to the composition has been seen to be a viable approach for enhancing some of their features. In the presence or absence of grape seed oil, two biopolymers (fenugreek galactomannan and xanthan gum) were synthesized, and the several characteristics of the resulting films were thoroughly examined. Data showed that composite films made of two hydrocolloids performed better than films made of just one hydrocolloid in terms of film thickness, water vapor permeability, oxygen permeability, mechanical properties, and thermal properties. The positive and acceptable interaction between two biopolymers was primarily responsible for this improvement. All films showed an amorphous or non-crystalline structure, according to the X-ray diffraction patterns. Additionally, the images from the scanning electron microscope showed that composite films made from two hydrocolloids and fenugreek galactomannan films. Film thickness and strain at break value increased by adding oil to the formulation. Contrarily, the moisture content and absorption, water vapor and oxygen permeability, opacity, and ultimate tensile strength values of the films decreased with the incorporation of oil [43].

Panneerselvam et al. synthesized film utilizing xanthan gum and chitosan with reinforced CuO@Ascorbic acid nanoparticles, separately. Both the films exhibited strong antibacterial activity and antioxidant behavior when compared to bare surfaces; however, xanthan gum-based film outperformed the chitosan-based film in terms of packaging properties. Overall results showed that xanthan gum-based packaging material have the potential to be employed as a barrier coating in biological food packaging due to scavenging activity as the IC50 value is 52.9 which exhibited high antioxidant property and would be more suitable for packaging of food items such as fresh vegetables and meat [44]. Table 1 illustrates the utilization of xanthan gum-based packaging for inhibiting microorganisms through antimicrobial action and extending the shelf life of various products.

## 5. Edible Food Coating/Packaging Utilizing Xanthan Gum

**Figure 7:**
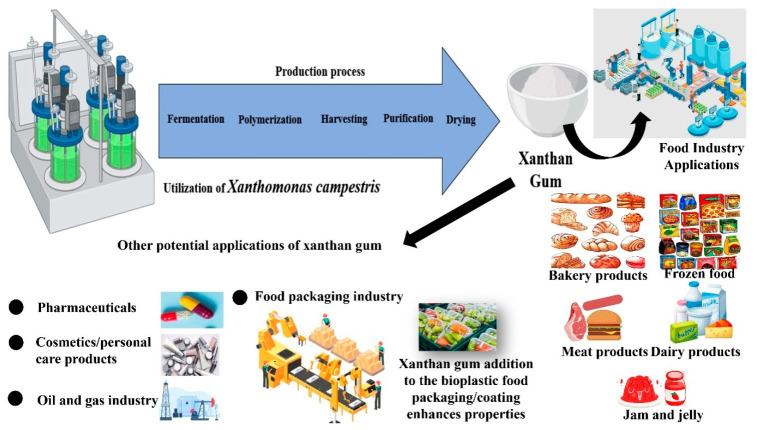
Production and applications of xanthan gum.

Researchers are trying to utilize xanthan gum in edible food coating or packaging due to its unique properties, such as enhancing the viscosity and texture of coatings, as it is a natural thickening and stabilizing agent. Its ability to form a flexible and transparent film makes it ideal for creating protective layers on food surfaces. Moreover, xanthan gum exhibits good barrier properties, preventing moisture loss and maintaining the freshness of the coated food. This biocompatible and edible polymer serves as an effective solution for developing environmentally friendly and sustainable food packaging, contributing to the preservation and quality of packaged products. The production and applications of xanthan gum is presented in Figure 7. An edible coating was synthesized by a group of scientists through casting, utilizing Plantago ovata seed mucilage, glycerol, and xanthan gum. The film became highly transparent and hydrophobic in nature, possessing low solubility and water vapor transfer rate. Surface morphology analysis revealed smooth surfaces, free of pores and fractures. It also displayed antioxidant and antibacterial property against *E. coli*, *S. aureus*, *P. aeruginosa*. Additionally, it prolonged the shelf life of strawberries to 8 days [45].

A brand-new edible coating was developed by Chen and associates, utilizing xanthan gum that contains nano-encapsulated *Litsea cubeba* essential oil for the preservation of salmon at 4 °C. First, the findings from scanning electron microscopy and growth curves demonstrated that the coating exhibited strong antibacterial activity against *V. parahaemolyticus*. By reducing salmon oxidation and limiting the growth of microbes (Total Viable Count and *V. parahaemolyticus*) within 8 days of storage at 4 °C, the coating successfully postponed the qualitative degradation of salmon [47]. With the aim of developing a high-performance edible packaging material for preserving freshly cut vegetables, tea polyphenols were added to the xanthan hydroxypropyl methylcellulose film forming solution to create the composite packaging material. The tensile strength and elongation at break were at their highest at the optimum condition. Its improved antioxidant and antibacterial qualities, effectively inhibiting *Staphylococcus aureus*. After 8 days, the packaging maintained 127.81% more vitamin C than unpackaged fresh-cut bell pepper. Additionally, compared to unpackaged, the melanodialdehyde concentration in green peppers was greater by 39.16%. The edible film manufactured by Mohsin and coworkers using curdlan and xanthan biopolymers showed that the xanthan and curdlan interacted strongly through an intermolecular hydrogen bond at pH 5. By maintaining self-aggregation of xanthan chains through hydrogen bonding at this pH, the xanthan-curdlan hydrogel was able to maintain the original structure of xanthan and create a strong intermolecular link with curdlan. Additionally, the blend films produced by the 5:5 and 4:6 ratios of xanthan and curdlan showed increased interaction, leading to their outstanding miscibility. The blend film reached a good amount of tensile strength, as required for its further practical application [57]. The effects of a xanthan gum based edible coating (2.5 g/L) applied either alone or enhanced with cinnamic acid (1 g/L) on the qualitative characteristics of freshly cut Asian pears (*Pyrus pyrifolia* L. cv. ‘Nashpati’) and European pears (*b* cv. ‘Babughosha’) stored at 4 °C. In contrast to fresh cut pears coated only with xanthan gum and uncoated pears, the addition of cinnamic acid as an antioxidant agent to xanthan gum based edible coating significantly (*p* ≤ 0.05) delayed the oxidative browning, decreased ascorbic acid level, degraded the content of total phenolics, and reduced antioxidant capacity. During the eight days of storage, the control slices of Nashpati showed a larger increase in browning index and polyphenol oxidase activity, as well as a constant fall in lightness values than those of Babughosha. Additionally, the decreased microbial growth on the fresh cut pear may be credited to the combined effects of favorable processing conditions and a cinnamic acid-enriched xanthan gum edible covering. Thus, authors claim the studied edible coating made of xanthan gum and cinnamic acid may help to prolong the shelf life of freshly cut Nashpati and Babughosha for 4 days and 8 days, respectively, at 4 °C by reducing surface browning [51]. One new type of well-developed edible composite film was made by a group of researchers utilizing pectin, sodium alginate, and xanthan gum. The maximum value of tensile strength was achieved 29.65 MPa. The resulting elongation at break was 19.02%, and a water vapor transfer rate of 18.12 × 10 11 g/(m^2^.s.pa). Furthermore, fresh cut potatoes were preserved using this nanocomposite in the form of coating/film in the shelf life studies [52].

Mango peel, often seen as waste, contains beneficial bioactive compounds for edible coatings or films. A study assessed phenolic mango peel extract effects on xanthan gum-based coatings. Solutions were stable with shear-thinning behavior and weak gel properties. Extracts did not change solution characteristics but influenced gel structure with temperature, making them suitable for complex food systems like coatings or delivery systems [58].

Another study explored using apple and rose as raw materials, adding xanthan gum and basil seed gum independently and in edible combinations. Results showed that adding gums increased hardness, gumminess, stiffness, and self-supporting ability. Higher xanthan gum levels lowered viscosity and yield stress [59]. A team of researchers investigated the impact of edible coatings containing gum arabic, carrageenan, and xanthan gum with 1% lemon grass essential oil on the postharvest quality of strawberries over 12 days of refrigerated storage. Results indicated that all three coatings maintained fruit quality parameters (including reduced weight loss, decay percentage, and enhanced retention of ascorbic acid, antioxidant activity, firmness, color parameters, as well as minimized enzyme activities related to fruit degradation) compared to the control group. They also exhibited antimicrobial properties against psychrophilic bacteria, yeast, and mold growth. The authors concluded that it is highly effective in extending the shelf life and maintaining the quality of strawberries during refrigerated storage for up to 12 days [46]. Joshi and associates presented a zinc oxide encapsulated xanthan-based edible coating with strong antibacterial properties. Biocompatibility tests demonstrated compatibility with blood cells and good cell viability. The hybrid coating significantly reduced weight loss in apples and tomatoes compared to uncoated samples under ambient conditions [49].

On tomatoes, the effects of an edible coating made of whey protein isolate, xanthan gum, clove oil, and glycerol monostearate were assessed by researchers Kumar and Saini. Investigations were conducted on the impact of coatings on several quality parameters at intervals of 3, 6, 9, 12, and 15 days after storage in a controlled setting (20 °C and 85% relative humidity). The stiffness and color of the coated samples improved after storage. The coated samples exhibited superior retention of the titrable acidity, ascorbic acid content, total phenolics content, total sugars, and reduce sugars in comparison to the non-coated sample. The outcomes showed that all prepared coatings had the ability to preserve quality attributes and lengthen tomatoes’ shelf life. The coatings prepared from whey protein isolate and xanthan gum in equal amounts showed the best combination of all the coatings since they minimized the conversion of starch into sugar and inhibited respiration, hence retaining optimum quality features. Because the coatings are edible, non-reactive, and biodegradable, their application has been claimed to be viable by the researchers [50]. Soleimani and team explored the use of xanthan gum and flaxseed mucilage as edible coatings for cheddar cheese over a 90-day ripening period. Different coatings were applied to cheddar cheese blocks, including polyvinyl acetate (control), 0.5% xanthan gum, and varying concentrations of flaxseed mucilage. The samples were stored at 8 ± 2 °C for 3 months. Results indicated changes in moisture and protein content during ripening, while pH, acidity, fat in dry matter, and other parameters were significantly affected by the coatings. Edible coatings showed a non-significant impact on total bacterial growth but significantly altered lactic acid bacteria growth [53].

Researchers investigated the impact of spraying xanthan gum-based coatings on fresh-cut lotus root for barrier and microbial properties. Solutions with varying xanthan gum concentrations, citric acid, and glycerol were sprayed onto lotus root slices and assessed for color, pH, morphology, and microbial counts during 16-day storage at 5 °C. The spray-coated samples displayed reduced color changes and inhibited Bacillus subtilis growth compared to non-coated samples, indicating enhanced shelf-life and microbial stability. The study suggests that xanthan gum-based spray coatings offer a promising approach to improve the storage stability of fresh-cut lotus root and may have broader applications for other perishable agricultural products [54]. Zambrano-Zaragoza and collaborators assessed the impact of β-carotene release rates from nanocapsules in xanthan gum coatings on fresh-cut cantaloupe melon. The nanocapsules reinforced xanthan gum treatment showed β-carotene release patterns resembling Higuchi-type behavior, indicating a controlled release. The nanocapsule incorporated xanthan gum treatment demonstrated minimal changes in whiteness and firmness, suggesting improved coating properties. Overall, it was found that this coating proved to be able to enhanced preservation, extending storage time up to 21 days at 4 °C [55].

Zamani and Farzaneh examined the influence of different temperatures (160 °C, 180 °C, and 190 °C) and hydrocolloid coatings (Basil seed gum, xanthan gum, methylcellulose, Basil seed gum-xanthan gum blend, and Basil seed gum-methylcellulose blends) on various properties of deep-fried potato strips in their research. It was observed that the coated samples showed slower oil absorption rates compared to the uncoated ones, with the basil seed and xanthan gum mixture (50:50) and exhibiting the lowest oil uptake at 0.14% dry basis, respectively. The effective moisture diffusivity was highest in uncoated samples and lowest in basil seed gum and xanthan gum blend coat. Increasing frying temperature accelerated oil uptake rates while reducing equilibrium oil content. Overall, basil seed gum and xanthan gum blend coated potato strips demonstrated promising results by absorbing less oil while maintaining similar organoleptic properties to the uncoated control [60].

## 6. Advanced Application of Xanthan Gum

Food industries adapt products and processes to meet consumer needs. Extruded snacks typically contain 10–20% fat for flavor retention. Another study suggests using xanthan gum as a fat substitute to reduce calorie intake in snacks and evaluated the impact of aqueous xanthan gum solutions (0.25%, 0.5%, 1.0%) at pH 3.5 and 7.0 on snack characteristics, including rheological properties and sensory attributes. Xanthan gum coatings exhibited favorable properties without altering texture or causing clumping. Sensory analysis showed high acceptability compared to oil-coated snacks, indicating that it is a suitable fat replacement in snack flavor coatings [61]. A previously published study assessed the impact of candeuba wax solid lipid nanoparticles and xanthan gum (coatings on guava, focusing on their effects on physicochemical and nutritional parameters. The concentrations of nanoparticles used (65 g/L and 75 g/L) were chosen based on their reported efficacy in post-harvest preservation, compared to xanthan gum coating and untreated samples. However, xanthan gum coat without nanofillers exhibited maturation rates of guava similar to control fruits [56]. In another instance, it was found that the blend of xanthan gum with chitosan slightly improved other characteristics; however, this was not significant and also the thermal stability dropped [3]. In films made by casting, the impact of partially substituting 10 and 20 weight percent of gellan, xanthan, or pullulan gums with cassava starch was examined. The starch films’ tensile behavior was enhanced by xanthan gum, but their water sorption capacity and water vapor permeability remained unchanged [62].

## 7. Mechanical Property Enhancement

Xanthan gum plays a crucial role in enhancing the mechanical performance of bioplastics, as is evident from the comprehensive data in several previously conducted studies on different composite materials. When xanthan gum was blended in chitosan matrices it improved tensile strength to some extent (from 3.67 to 5.68 MPa). Polyelectrolyte formation between two opposite charged biopolymer resulted in increased interaction and enhanced strength; however, it reduced flexibility and transparency [3]. Similar observation was also observed in other studies [29]. Although there was a reduction in elongation at break, the overall enhancement in tensile strength signifies the reinforcing capabilities of xanthan gum. Moreover, the incorporation of xanthan gum in blends with curdlan and gelatin further amplifies the mechanical properties of bioplastics. The xanthan and curdlan blend, especially in the ratio of 2:2:6 with gelatin, exhibits an impressive tensile strength of 38.22 MPa and an elongation at break of 18.92%, showcasing the synergistic effects of these components [63]. Furthermore, the versatility of xanthan gum is highlighted in its role as a crosslinking agent in gelatin-based nanofibers, resulting in a remarkable ten-fold increase in tensile stress compared to the control [41]. In the creation of an edible composite film using pectin, sodium alginate, and xanthan gum, the maximum tensile strength reached 29.65 MPa [52]. A notable increase in the tensile strength of agar was achieved to 28.9 MPa from 25 MPa and further to 40 MPa upon the addition of xanthan gum. The incorporation of xanthan gum also led to a significant improvement in the elongation at break of the composites. However, it is worth noting that the introduction of xanthan gum in higher amounts could potentially introduce disruptions in the polymer matrix, potentially limiting the stretching capability. So, optimization research is essential here. The agar–xanthan gum composite (agar 3 g + xanthan gum 1 g) exhibited a significant increase (statistically significant at *p* < 0.05) in both tensile strength and elongation at break. This improvement was attributed to a higher degree of crosslinking reactions facilitated by an increased percentage of both agar and xanthan gum. Xanthan gum, known for its heteropolysaccharide nature and substantial molecular weight, played a role in enhancing the mechanical properties of the composite films [42]. In the xanthan-curdlan gum blend fabricated for the edible food packaging, it has been observed that a high tensile strength was achieved in the 5:5 and 4:6 xanthan gum/curdlan gum films, with the maximum tensile strength reaching 28.13 MPa in the 5:5 blend. As the blending ratio of both polymers increased, the tensile strength of the blended films improved, reaching a peak of 5:5. This noteworthy improvement can be attributed to enhanced interactions, including hydrogen bonding, electrostatic forces, and van der Waals forces, resulting from the combination of these polymers. The increased hydrogen bonding interactions between xanthan and curdlan molecules are thought to contribute to the heightened the strength in the blended films. Consequently, it can be inferred that blending xanthan gum to different polymers proves to be an effective strategy for enhancing the mechanical properties of films [58]. Researchers also tried combining xanthan gum with the pullulan and grape seed extract and find out, the strength reaching 16.62 MPa and flexibility to reach 22%. Overall, the data underscores xanthan gum’s significance in reinforcing bioplastic materials, showcasing its multifaceted role in enhancing the mechanical capabilities and structural integrity of these sustainable alternatives. Table 2 displays various xanthan gum-based composites and their mechanical properties.

Films made of gelatin, carboxymethyl cellulose, and xanthan gum were casted by a Nur and the research team in order to evaluate the effects of their different ratios. The thickness, moisture content, and water vapor permeability of the gelatin carboxymethyl cellulose xanthan gum film was all increased by the addition of xanthan gum (*p* ≤ 0.05). Additionally, thermal stability (Tg) and UV light shielding also increased, coupled with decreasing visible light transparency (*p* ≤ 0.05) and increasing thermal stability (Tg) (*p* ≤ 0.05). However, it was observed that the produced films showed lower tensile strength with reduced elongation at the break point, higher puncture force, and lower puncture deformation, indicating higher puncture resistance than the control film [64].

## 8. Surface Morphology

**Figure 8:**
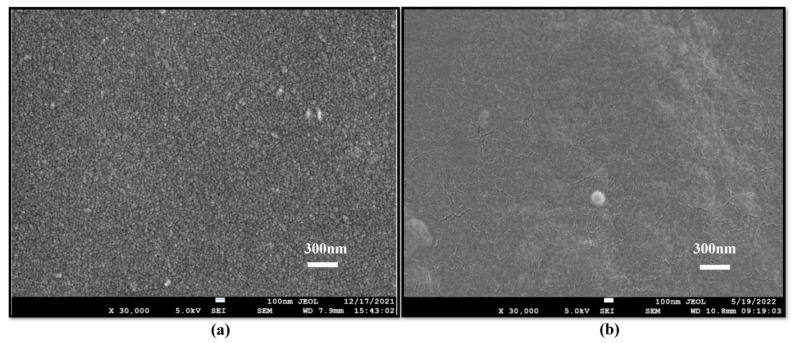
Smoother surface due to blend of xanthan gum [(**a**) chitosan (**b**) blend of xanthan gum in chitosan] [3,13].

Understanding the surface morphology of a packaging material is essential for optimizing its functionality and enhancing its performance, in its applications. The analysis of its surface structure provides insights into its texture, uniformity, and the distribution of any incorporated additives or fillers. Advanced characterization techniques such as Scanning Electron Microscopy (SEM), Field Emission Scanning Electron Microscopy (FE-SEM), Transmission Electron Microscopy (TEM) and Atomic Force Microscopy (AFM), etc., are commonly employed to study the surface morphology of biofilms. The literature in this field suggests that morphological analysis of xanthan gum-based films revealed smooth, uniform surfaces devoid of pores and cracks [45]. Micrographs from Scanning Electron Microscopy illustrate that films incorporating TiO_2_–Ag and xanthan gum exhibit a homogeneous structure with a granular texture [36]. In one of our prior published experimental work, Field Emission Scanning Electron Microscopy (FE-SEM) demonstrated that the addition of xanthan gum to chitosan resulted in a smoother surface compared to the rough texture of pure chitosan surface [3], as depicted in Figure 8. De Morais Lima and research group also found that the xanthan gum-based films exhibited a uniform structure without phase separation or fissures, indicating that the polymers interacted effectively with each other, resulting in a cohesive and continuous matrix.

## 9. Thermal Property

Thermal analysis is necessary for packaging materials to determine their thermal stability, decomposition temperatures, and overall suitability for various storage and environmental conditions, ensuring product safety and performance. Thermal analysis can be performed using various techniques, such as Thermogravimetric Analysis (TGA), which measures the mass change in a substance as it undergoes a controlled temperature program. TGA enables the identification of specific temperature ranges where the sample attains a fixed chemical composition and tracks the progression of reactions such as dehydration, oxidation, combustion, and decomposition. A research investigation showed that xanthan gum films exhibited weight loss up to 120 °C, attributed to the water absorbed in the films. The onset of thermal decomposition of organic matter was observed from 120 °C, with no significant variations among different composite ratios. The addition of xanthan gum did not affect the decomposition process. However, the presence of xanthan gum resulted in films with multiple stages of thermal decomposition of organic matter, indicating chemical interactions among the materials used [29]. In another experiment, xanthan gum-based biopolymer blend proved to have high degradation rate; however, when nanoparticles were reinforced, a subtle upgrade was observed. At the end, at 499.28 °C it was left with a 45.74 weight %. Better miscibility among the matrix and fillers can be the reason for this slight enhancement in stability [3]. In a composite consisting of xanthan gum and sodium montmorillonite clay (Na^+^MMT), thermogravimetric analysis demonstrated that the clay enhanced the thermal stability of the aerogels. However, when xanthan gum was blended with agar, the thermal stability deteriorated. Flammability tests using a cone calorimeter indicated that xanthan gum/clay aerogels exhibited lower flammability compared to other common foams. The clay acted as a barrier to heat and mass transfer, significantly boosting the flame retardancy of the base aerogels [35]. In case of lemon peel powder containing xanthan gum and TiO_2_–Ag nanoparticles, the TGA spectra revealed that all the film samples undergone weight loss in two stages. The initial weight loss, which occurred between 80 °C and 130 °C, attributed to the loss of water and surface evaporation of moisture from the films, corresponding to the amount of water absorbed in the biopolymer. Films incorporating xanthan gum and nanoparticles demonstrated improved thermal stability compared to the control sample, due to the interaction between all the materials [36]. A similar finding was observed in another study of gellan gum film containing xanthan gum/TiO_2_–Ag nanoparticles [65]. The glass transition temperature (T_g_) and melting temperature (T_m_) of the agar/xanthan gum composite films showed a slight improvement compared to pure agar. Thermogravimetric analysis (TGA) indicated a significant increase in char yield and enhanced thermal stability [42]. Gelatin-carboxymethyl cellulose-xanthan gum films were also found to have increased thermal stability (*p* < 0.05) [64].

## 10. Antioxidant Property

Antioxidant properties in food packaging and coatings are crucial for extending the shelf life and preserving the quality of food products. Antioxidants help prevent oxidative degradation, a process that can lead to the spoilage of food, resulting in rancidity, color changes, off-flavors, and loss of nutritional value. This oxidative degradation is primarily caused by the exposure of food to oxygen, light, and heat, which can trigger the formation of free radicals and subsequent oxidation of fats, oils, and other sensitive food components [66]. The literature suggests that low-molecular-weight xanthan gum has demonstrated antioxidant properties and a protective effect on H_2_O_2_-injured Caco-2 cells, indicating its potential use in various fields to combat oxidative damage caused by excessive reactive oxygen species [67]. Xanthan gum is an effective inhibitor of oil peroxidation and shows antioxidant activity for human corneal epithelial cells. Xanthan gum also exhibits DPPH free radical scavenging activity after carboxymethylation [68]. When oxidized in an alkaline medium, xanthan gum showed enhanced antioxidant activity compared to its native form. Conversely, xanthan gum oxidized under acidic conditions but with a similar molecular weight had relatively lower antioxidant activity [69].

## 11. Barrier Property

Barrier properties are essential in packaging to maintain product quality by preventing the ingress of oxygen, moisture, and contaminants. They extend the shelf life of perishable goods, protect against external contaminants, and maintain the aesthetic and functional integrity of products. Good barrier properties also ensure compliance with industry regulations, enhance consumer confidence, and improve cost efficiency by reducing spoilage and the need for additional preservatives. The rate at which water vapor passes through the film is crucial in determining the shelf life of packaged food. Food can quickly spoil due to moisture absorption from the environment. Therefore, water resistance is vital for ensuring film stability and preventing the microbiological decay of stored food. Thus, the packaging film must possess an effective moisture barrier. Due to its natural hydrophilicity, the pure chitosan film exhibited the highest water vapor transmission rate (WVTR). The chitosan–xanthan gum blend, which is moderately hydrophilic, showing about 11.88% less water vapor permeability. This reduction is attributed to the interaction between the biopolymers and the formation of hydrogen bonds. Although it is not ideal to completely prevent gas permeability for fresh fruits and vegetables due to their cellular respiration, excessive oxygen inside packaging can reduce food shelf life by creating conditions favorable for microbial growth. Oxygen transmission can lead to oxidation of lipids and vitamins, negatively impacting sensory and nutritional qualities, as well as altering food properties such as taste and color. Using nanocomposite packaging materials with a suitable oxygen barrier can enhance food quality and extend shelf life. The oxygen transmission rate (OTR) through packaging films depends on factors like microstructure, void volume, and the structural arrangement of polymer chains. Strong interactions and an organized hydrogen bonding network can reduce OTR and oxygen permeability (OP) values.

## 12. Impact of Structural Configuration on Xanthan Gum Composite Performance

The recent literature demonstrates a strong correlation between FTIR- and XRD-derived structural signatures and the functional output of xanthan gum-based composites. For example, FTIR spectra frequently reveal shifts in O–H, C=O, and glycosidic bands when xanthan is blended with other biopolymers or inorganic fillers, indicating hydrogen bonding and electrostatic complexation, which in turn enhance tensile strength and barrier properties. In a study by Tabassum et al., the incorporation of montmorillonite nanoclay into a chitosan–xanthan blend yielded FTIR evidence of strong interfacial interactions, while XRD showed reduced crystallinity; these structural changes correlated with significantly improved oxygen and moisture barrier behavior in the films [13]. Similarly, in a ZnO-reinforced chitosan–xanthan nanocomposite, FTIR confirmed strong coordination bonding and polyelectrolyte complex formation, while XRD patterns demonstrated well-dispersed ZnO phases coexisting with the polymer matrix—this morphological integration resulted in enhanced mechanical strength, UV protection, and biodegradability [3]. Further, in alginate/xanthan films reinforced with halloysite nanotubes loaded with ZnO and plant extract, FTIR and XRD analyses validated the successful incorporation and interaction of nanohybrids, which contributed to increased tensile strength, thermal stability, and lower water vapor permeability [70]. Additionally, green-synthesized ZnO@xanthan gum nanocomposites exhibited distinct XRD peaks for ZnO alongside modified polysaccharide crystallinity, while FTIR confirmed surface functionalization; this structural synergy underpinned strong biofilm inhibition activity and antimicrobial efficacy [71]. Collectively, these mechanistic insights from spectroscopic and diffractometric studies underscore that molecular-level interactions in xanthan gum composites are directly responsible for their improved mechanical, barrier, and functional properties, making FTIR and XRD indispensable tools for designing high-performance biopolymer packaging materials.

## 13. Resource Efficiency Angle

According to various scientist including Asase and Glukhareva xanthan gum is a commercially significant biopolymer with broad applications across food, pharmaceutical, and industrial sectors, largely due to its unique rheological and stabilizing properties. However, its production remains cost-intensive, primarily because glucose serves as the predominant carbon source, contributing up to 70% of the total raw material cost. Recent studies have explored the use of food and agro-industrial wastes, such as olive pomace [72], cheese whey [73], wheat bran [74], as low-cost and sustainable alternatives, demonstrating potential reductions in production costs of 60–70%. Xanthan synthesized from these waste substrates exhibits yields and thermal stability comparable to conventional commercial gum; nevertheless, variations in structural and functional characteristics have been observed, necessitating stringent quality control measures [75,76]. This strategy offers the dual benefit of reducing production costs while advancing waste valorization and environmentally sustainable manufacturing practices [77,78,79]. Waste-derived xanthan gum or other waste-derived compounds combined with xanthan gum can be further utilized to develop sustainable packaging and coatings, promoting circular economy practices, resource efficiency, and environmental sustainability.

Moist olive pomace (MOP), a high-moisture by-product of olive oil production, poses handling challenges that necessitate sustainable valorization strategies. A study explored xanthan gum (XG) biosynthesis using MOP (0–50%) as a substrate additive to induce bacterial stress. The resulting XG was evaluated for yield, structure (FTIR), thermal stability (TG), rheology, and antioxidant activity. Incorporation of up to 30% MOP significantly enhanced XG yield, with 15% MOP achieving a 50.9% increase in production and nearly fourfold higher viscosity. MOP-enriched XG also exhibited notable antioxidant properties, adding functional value for food, pharmaceutical, and cosmetic uses [72]. Grape (*Vitis vinifera*) pomace, rich in soluble carbohydrates, was evaluated as a sole carbon source for xanthan gum (XG) production by four Xanthomonas strains. Fermentation kinetics, gum yield, and rheological properties were assessed, with the produced XG further tested in a pudding model. The *X. axonopodis* pv. *vesicatoria* strain yielded up to 5.1 g/L XG 6.25% higher than the standard strain while *X. hortorum* pv. *pelargonii* exhibited superior viscosity (46 Pa·s at 1%, 14 Pa·s at 2%). Although commercial gum produced the highest pudding viscosity (254.7 Pa·s), grape pomace supported efficient XG synthesis without pretreatment, demonstrating its potential as a cost-effective and sustainable substrate for industrial biopolymer production [80]. Another study focused on optimizing xanthan gum (XG) production by X. campestris using diverse carbon, nitrogen, and renewable sources. Tested substrates included sugars (glucose, fructose, sucrose, etc.), amino acids and peptones, and agro-wastes such as citrus and fruit peels. The highest XG yield (32.34 g/L) was obtained with L-glutamic acid after 72 h fermentation, while renewable substrates achieved comparably high yields within 24 h. Gums derived from renewable sources also exhibited superior antioxidant activity (DPPH and reducing power assays). Structural confirmation by FTIR and ^1^H NMR verified typical XG features. Overall, the study demonstrates a sustainable and efficient approach for producing antioxidant-rich xanthan gum using renewable bioresources [81]. Liquid pineapple waste (LPW), an industrial by-product, as an alternative carbon source for xanthan gum was assessed for its production by X. campestris ATCC 13951. Various carbon sources, including glucose, were tested, and fermentation efficiency was evaluated through cell dry weight and xanthan yield over 96 h. LPW achieved a xanthan yield of 5 g/L, demonstrating its viability as a low-cost substrate. A concentration of 60 g/L LPW was identified as optimal for effective bioprocess performance [82]. Hence, xanthan gum can potentially be produced from waste materials or blended with waste-derived compounds. These materials are biodegradable, non-toxic, and environmentally friendly (Table 3).

## 14. Carbon-Neutral Packaging Potential

Reducing carbon footprint is essential for mitigating climate change, and conventional plastics remain major contributors due to their petrochemical origin and persistent non-biodegradability. In contrast, xanthan gum-based packaging naturally de-grades, reducing landfill burden, and preventing environmental pollution. Substituting traditional plastics with xanthan gum materials can significantly lower greenhouse gas emissions across the product lifecycle, offering a more sustainable and eco-friendly packaging alternative.

In our previously published experimental research, we have reported biodegradability of xanthan gum–chitosan blend and its nanocomposites. When xanthan gum was blended with biopolymer like chitosan it degraded approximately 70% in the first month and 100% in the second; in natural soil after discarding. The most durable xanthan gum-based nanocomposite containing 3 wt% of ZnO nanoparticles exhibited a weight loss of 88% at the end of the second month [3]. A xanthan gum blend with polyvinyl alcohol–xanthan gum completely decomposed in soil and water within 12 h, which was not only significantly shorter than commercial plastic bag, but also other biodegradable materials [38]. As a result, adopting xanthan gum-based sustainable packaging not only addresses immediate environmental concerns but also supports long-term sustainability goals by promoting a circular economy where materials can be safely returned to the natural environment without causing harm. Several studies have claimed that xanthan gum-based packaging is biodegradable, primarily because xanthan gum itself is biodegradable, and the additional materials used in those studies are also biodegradable. However, these claims are often made without performing degradation studies. So, these types of experimentation are recommended to ensure their lower carbon footprint for sustainability in the environment. Using renewable sources such as waste products as raw materials for sustainable biodegradable or compostable packaging can significantly contribute to a carbon negative footprint by reducing the reliance on fossil fuels and minimizing carbon emissions. This approach also helps sequester carbon that would otherwise be released from waste decomposition, while promoting the recycling of resources. It can reduce landfill usage and lower greenhouse gas emissions. This process not only sequesters carbon but also supports a circular economy by creating value from waste and promoting resource efficiency. Moreover, such practices foster industrial symbiosis, where the by-products of one industry become the raw materials for another, enhancing overall sustainability and reducing environmental impact. This holistic approach integrates waste management, renewable resource utilization, and sustainable production, driving the transition towards a greener and more resilient economy [5,91]. Waste-derived xanthan gum or other waste-derived compounds that are combined with xanthan gum can be further utilized to develop sustainable packaging and coatings, supporting SDGs 9, 12, and 13 through waste valorization and eco-friendly innovation.

## 15. Conclusions

Biopolymers have been the subject of extensive research recently, particularly regarding their potential applications in packaging. This presents an opportunity for their widespread adoption due to their eco-friendly and reliable characteristics. However, challenges persist in commercializing these materials, mainly due to limitations in their barrier properties, thermal stability, and mechanical strength. This review emphasizes the significant role of xanthan gum in advancing the realm of bioplastics for food packaging and coatings, offering a sustainable and economically viable alternative to traditional plastics. The insights provided pave the way for future research and industrial applications, fostering the development of high-performance and environmentally conscious options. Xanthan gum has emerged as a versatile and sustainable biopolymer with significant potential to transform food preservation and packaging technologies. Its unique rheological, film-forming, and stabilizing properties enable it to serve as an effective matrix material or functional additive in the formulation of biodegradable packaging films and edible coatings. Recent advancements have demonstrated that xanthan gum, when blended or reinforced with other biopolymers, nanoparticles, or natural extracts, can substantially enhance the mechanical strength, barrier performance, and antimicrobial activity of bio-based films. Such improvements contribute directly to extending the shelf life of perishable products while reducing dependence on conventional, non-biodegradable plastics. Right now, two main challenges associated with the use of xanthan gum in bioplastic modifications are as follows; first, high concentrations of xanthan gum can cause structural disruptions within the polymer matrix, compromising its stretchability. Therefore, it becomes crucial to optimize the blend composition to strike a balance between the beneficial effects of xanthan gum and its potential drawbacks, ensuring the bioplastic’s performance, remains suitable for practical applications. The second point is the high cost of xanthan gum production from synthetic media which represents another challenge. To mitigate this, various approaches are being explored, including the utilization of waste sources to reduce raw material and overall costs. Strategies such as adjusting nutrient content, optimizing feeding methods, controlling temperature and pH, enhancing agitation, and incorporating antifoam agents in xanthan gum fermentation processes have been investigated to make production more cost-effective. The integration of waste-derived or bio-based xanthan gum aligns with sustainable development goals by promoting waste valorization, resource efficiency, and environmental protection. These innovations highlight xanthan gum’s dual role as both a performance enhancer and a sustainability driver within modern food preservation systems. Moreover, its compatibility with renewable fillers, bioactive compounds, and nanomaterials expands its application potential across a range of food products and environmental conditions. Despite these advancements, challenges remain regarding large-scale production, cost reduction, and the optimization of film-forming formulations to achieve industrial viability. Future research should focus on developing greener synthesis and modification methods, exploring synergistic polymer–nanofiller interactions, and evaluating long-term storage stability and biodegradation behavior under real-world conditions.

## Figures and Tables

**Table 1:** Xanthan gum-based composite packaging and their properties.

S. No.	Composite	Microorganisms Inhibited	Shelf-Life Extension	Reference
1.	Edible coating made of *Plantago ovata* seed mucilage, glycerol, and xanthan gum	*E. coli*, *S. aureus*, *P. aeruginosa*	Prolonged shelf life of strawberries till 8 days	[45]
2.	Apple, rose, xanthan gum, basil seed gum in edible combinations	-	Maintained quality of strawberries over 12 days of refrigerated storage	[46]
3.	Edible coating made of xanthan gum that contains encapsulated *Litsea cubeba* essential oil	*V. parahaemolyticus*	Preserved salmon at 4 °C.	[47]
4.	Xanthan gum and TiO_2_ Ag nanoparticles, lemon peel powder film	*E. coli* and *S. aureus*	-	[36]
5.	Xanthan gum and hydroxypropyl methylcellulose blend	-	Prolonged shelf life of banana	[37]
6.	Tea polyphenols, xanthan hydroxypropyl methylcellulose tea polyphenols composite	*S. aureus*	Prolonged green bell pepper shelf life till 8 days	[48]
7.	Pullulan, xanthan gum with grape seed extract	*Bacillus subtilis*, *Escherichia coli*, and *Staphylococcus aureus*	Prolonged shelf life of fresh cut apples	[39]
8.	Zinc oxide encapsulated xanthan-based edible coating	-	Maintained good quality of apples and tomatoes under ambient condition	[49]
9.	Edible coating made of whey protein isolate, xanthan gum, clove oil, and glycerol monostearate	-	Prolonged shelf life of tomatoes	[50]
10.	Extracts from *Polygonatum cyrtonema*, xanthan gum, flaxseed gum, and carboxymethyl cellulose	-	Prolonged shelf life of mango	[40]
11.	Xanthan gum based edible coating with cinnamic acid	-	Prolonged shelf life of freshly cut “Nashpati” and “Babughosha” for 4 days and 8 days, respectively, at 4 °C	[51]
12.	Edible composite made of pectin, sodium alginate, and xanthan gum	-	Maintained quality of fresh cut potato	[52]
13.	Xanthan gum and flaxseed mucilage as edible coatings	-	The shelf life of cheddar cheese blocks for 3 months while stored at 8 ± 2 °C	[53]
14.	Xanthan gum, citric acid, and glycerol	*Bacillus subtilis*	Extended shelf life of lotus root slices for 16 days storage at 5 °C	[54]
15.	Nanocapsules in xanthan gum coatings	-	Enhanced preservation of fresh-cut cantaloupe melon, extending storage time of up to 21 days at 4 °C	[55]
16.	Candeuba wax solid lipid nanoparticles and xanthan gum coatings		Xanthan alone is not able to extend the shelf life, whereas, incorporation of the nanoparticles improves this ability	[56]

**Table 2:** Xanthan gum-based composites and their mechanical capabilities.

S. No.	Composites	Mechanical Capabilities	Reference
1.	Xanthan gum-chitosan blend	Tensile strength enhancement from 3.67 to 5.68 MPa, but elongation at break reduced.	[3]
2.	Xanthan gum-chitosan blend	Tensile strength 14.07 MPa and elongation at break of 9.03%	[29]
3.	Xanthan gum-chitosan-hydrolysate	12.19 MPa of tensile strength and elongation of 9.56%	[29]
4.	Xanthan and curdlan blend (5:5)	Maximum tensile strengths of 28.13 MPa	[58]
5.	Xanthan and curdlan blend (4:6)	Maximum tensile strengths of 26.45 MPa	[58]
6.	Xanthan, curdlan, gelatin (2:2:6)	Tensile strength of 38.22 ± 0.7 MPa, along with the highest elongation at break of 18.92 ± 0.5%	[63]
7.	Pullulan polysaccharide, xanthan gum with grape seed extract	Tensile strength of 16.62 ± 1.27 MPa and elongation at break of 22.60 ± 0.48%	[39]
8.	Gelatin-based nanofibers and oxidized xanthan gum as a crosslinking agent	Tensile stress of 13.24 ± 0.76 MPa, 10 times more than the control	[41]
9.	Agar and xanthan gum composite	The tensile strength ranged from 25 to 40 MPa and elongation at break ranged from 28.9 to 39.4%	[42]
10.	Edible composite film utilizing pectin, sodium alginate, and xanthan gum	The maximum value of tensile strength was achieved 29.65 MPa	[52]

**Table 3:** Biobased or waste-derived ingredients in xanthan gum films, coatings, and composites, with preparation, and application outcomes in food packaging.

S. No.	Biobased/Waste-Derived Ingredient(s)	Material (Film/Coating/Composite)	Preparation/Key Components	Food Tested/Key Outcome	Reference
1.	*Opuntia ficus*-*indica cladodes* powder (plant biomass) blended with gum arabic + xanthan	Biocomposite film	Casting of cladodes powder + gum arabic + xanthan; dried films	Edible biocomposite films with promising mechanical and barrier properties for food packaging applications	[83]
2.	(Biobased carrier) xanthan gum as matrix for probiotic cells (*L. plantarum* 75)	Edible coating (xanthan carrying probiotics)	Aqueous xanthan coatings loaded with probiotic culture; applied to fresh-cut melons	Coating retained viable probiotics, reduced postharvest losses and preserved antioxidant properties of fresh-cut cantaloupe and honeydew	[84]
3.	Xanthan gum (as a natural polysaccharide coating) possibly combined with other bioactives	Edible coating	Aqueous xanthan coatings applied to fruit surfaces	Xanthan coatings helped preserve postharvest quality of guava (slowed physiological degradation), extending shelf life	[85]
4.	Xanthan gum + essential oils (clove, cinnamon) natural antimicrobials	Edible coating	Xanthan-based coating enriched with essential oils; cold-storage study	Xanthan + essential oil coatings extended pomegranate shelf life under cold storage and improved microbial/quality metrics	[86]
5.	Gelatin (protein) blended with xanthan (biopolymer) both biobased	Composite edible film	Solution casting of gelatin blended with xanthan (and other cellulose derivatives)	Composite films showed improved mechanical and biological properties versus neat gelatin suitable as ecofriendly packaging films	[87]
6.	Hydroxypropyl guar gum + xanthan + curcumin + ZnO NPs	Hydrogel film	Crosslinked with citric acid, loaded with curcumin and ZnO nanoparticles	Biodegradable (30 days) film used for strawberry coating antimicrobial and antioxidant activity	[88]
7.	Xanthan + carboxymethyl cellulose + green-synthesized ZnO (from coriander extract)	Composite coating	Green ZnO synthesis + polymer blending	Active packaging film with enhanced antimicrobial and UV-barrier performance	[89]
8.	Pomelo peel extract (citrus waste) + xanthan nano emulsion	Edible coating	Nano emulsion of peel extract incorporated into xanthan matrix	Applied on paneer; reduced microbial growth and improved shelf life	[90]

## Data Availability

No new data were created or analyzed in this study. Data sharing is not applicable to this article.
